# Twists through turbidity: propagation of light carrying orbital angular momentum through a complex scattering medium

**DOI:** 10.1038/s41598-024-70954-x

**Published:** 2024-09-05

**Authors:** Fatima Khanom, Nawal Mohamed, Ivan Lopushenko, Anton Sdobnov, Alexander Doronin, Alexander Bykov, Edik Rafailov, Igor Meglinski

**Affiliations:** 1 College of Engineering and Physical Sciences, Aston University, Birmingham, B4 7ET UK; 2 Optoelectronics and Measurement Techniques, University of Oulu, P.O. Box 4500, 90014 Oulu, Finland; 3 School of Engineering and Computer Science, Victoria University of Wellington, Wellington, 6140 New Zealand

**Keywords:** Vortex beams, Orbital angular momentum, Light propagation, Turbid scattering medium, Twist of light, Optical physics, Optical physics

## Abstract

We explore the propagation of structured vortex laser beams-shaped light carrying orbital angular momentum (OAM)-through complex multiple scattering medium. These structured vortex beams consist of a spin component, determined by the polarization of electromagnetic fields, and an orbital component, arising from their spatial structure. Although both spin and orbital angular momenta are conserved when shaped light propagates through a homogeneous, low-scattering medium, we investigate the conservation of these angular momenta during the propagation of Laguerre–Gaussian (LG) beams with varying topological charges through a turbid multiple scattering environment. Our findings demonstrate that the OAM of the LG beam is preserved, exhibiting a distinct phase shift indicative of the ‘twist of light’ through the turbid medium. This preservation of OAM within such environments is confirmed by in-house developed Monte Carlo simulations, showing strong agreement with experimental studies. Our results suggest exciting prospects for leveraging OAM in sensing applications, opening avenues for groundbreaking fundamental research and practical applications in optical communications and remote sensing.

## Introduction

Navigating through complex media presents significant challenges in optical imaging and information transmission, primarily due to the erratic scattering of light^1^. Nano-scale spatial and/or temporal variations in the refractive index within complex random, heterogeneous, emulsions, powders, porous, polycrystalline materials and fractal media, such as biological tissue, multi-mode fibers, and atmospheric phenomena like clouds and fog lead to optical scattering, which distorts the wavefront of incident light^2^. The endeavor to elucidate, comprehend, and ultimately forecast the propagation of waves presents a formidable set of challenges to researchers within this domain. Since the inception of this field in the early twentieth century, it became apparent that the introduction of novel concepts and significant approximations was imperative. Noteworthy among these conceptual advancements are the effective medium theory^3,4^ and the radiative transport theory^5,6^, which remain pivotal to contemporary investigations. A principal challenge associated with these approximative frameworks arises from the often comparable scales of inhomogeneities within the complex medium to the wavelength of interest. These theories are primarily applicable in scenarios where the inhomogeneities’ length scales significantly exceed the wavelength. Consequently, numerous relevant cases emerge where the effective medium theory, radiative transport theory, or both may not adequately apply, leading to considerable discrepancies in their predictive capabilities.

In recent years, research efforts have concentrated on mitigating the impact of scattering to increase the penetration depths of light in turbid media^7^. Significant progress has been made in the understanding and application of Optical Reciprocity (OR) principles^8^. This advancement has facilitated the development of innovative techniques for focusing light deeply within tissue-like scattering media^9,10^ and unveiled the OR relationship between incident probing light and back-scattered light in turbid tissue-like medium.

Wavefront shaping emerges as a promising technique, offering precise control over the phase and amplitude of light waves. By manipulating light wavefronts, it becomes possible to shape the spatial distribution of light intensity effectively^11^. Recent advancements in optical wavefront shaping and phase recording techniques have significantly enhanced our capability to overcome the challenges posed by random light scattering in turbid media^12^. Traditional methodologies, including the diffusion equation, frequently failed to account for the complex interference patterns of scattered light, thereby exacerbating the challenge. Nevertheless, the advent of innovative approaches, such as optical wavefront shaping, precise phase recording, the employment of structured light^13,14^, and the application of complex optics, have effectively surmounted these historical limitations. These developments represent a paradigm shift in our approach to manipulating and controlling light in highly scattering environments, opening new avenues for research and application in fields ranging from biomedical imaging to optical communication^1^. These breakthroughs enable researchers to exert control over the coherent transport of light within highly scattering media, facilitating significant progress in various applications. Improved focusing and transmission of light through complex systems and the performance of intricate tasks within turbid environments, such as optical micro-manipulation, are now achievable. The capability to manipulate and control light waves in turbid environments marks a significant achievement in the field of optical engineering, heralding a wide range of practical applications and scientific breakthroughs. Techniques such as wavefront shaping, phase-conjugation, and the employment of spatial light modulators (SLMs) are instrumental in effectively directing the propagation of light^15^. The shaped light with a helical wavefront, possessing Orbital Angular Momentum (OAM)^16^ and also referred to as vortex beams (VBs)^17^, presents new unique opportunities in the field of biological imaging.

The OAM carried by vortex beams represents a novel set of carrier signals distinct from amplitude, phase, polarization, and frequency. When combined with traditional multiplexing methods, OAM can significantly enhance channel transmission capacity. Research has revealed that partially coherent vortex light fields, possessing both OAM and partial coherence, offer unique benefits in applications such as beam shaping, ghost imaging, optical communications, and information encryption^13,18^. Recent research underscores that OAM beams achieve superior penetration depths through complex, tissue-like scattering media compared to conventional Gaussian beams^16,19–21^. This attribute is highly advantageous for biomedical applications, where deeper penetration is crucial for achieving enhanced imaging resolution within deep tissue structures.

In current paper, we investigate the predictive capabilities of OAM shaped light associated with Laguerre-Gaussian (LG) beams^22^. This is achieved by analyzing the alterations in their helical wavefront as they propagate through a complex scattering medium, utilizing a specially developed Monte Carlo (MC) method presented below. The study encompasses various wave properties of light, including coherence, polarization, interference, and the phenomena of reflection and refraction at the boundary of the medium. These properties facilitate a comprehensive interpretation of light behavior within complex tissue-like scattering medium.

## Results

For LG beams, we rely on the following representation valid in paraxial approximation^23–25^:

1 $$\begin{aligned} \begin{aligned}L G_{p}^{\ell }(\rho , \phi , z)&= \sqrt{\frac{2 p !}{\pi (|\ell |+p) ! {w}^{2}(z)}}\left[ \frac{\sqrt{2} \rho }{{w}(z)}\right] ^{|\ell |} L_{p}^{|\ell |}\left[ \frac{2 \rho ^{2}}{{w}^{2}(z)}\right] \exp \left[ -\frac{\rho ^{2}}{{w}^{2}(z)}\right] \times \\& \quad \times \exp [i(2 p+|\ell |+1) \arctan (z/z_R)] \exp \left[ \frac{-i k \rho ^{2}z}{2(z^2+z_R^2)}\right] \exp [-i \ell \varphi ] \exp [-i kz]. \end{aligned} \end{aligned}$$

Here, $k=2\pi /\lambda$, ${w}(z)={w}(0) \sqrt{1+(z/z_R)^2}$, $z_R=\pi {w}^2(0)/\lambda$, *w*(0) corresponds to the zero-order Gaussian beam waist and is adjusted to fit experimental image, $\{\rho ,\phi ,z\}$ is cylindrical coordinate system with beam propagating along *z* axis. Then phase evolution of the LG beam is defined as^25^:

2 $$\begin{aligned} \Psi (\rho , \phi , z) = \arg \left( L G_{p}^{\ell }(\rho , \phi , z)\right) = \frac{-k \rho ^2 z}{2(z^2+z_R^2)} - \ell \phi - kz + G(z), \end{aligned}$$

where $G(z)=(2p+|\ell |+1)\arctan (z/z_R)$ corresponds to the Gouy phase. The helical phase of LG beam, characterized by a phase front that spirals around the beam’s axis, results from the azimuthal phase term in the beam’s electric field expression. This term correlates with the beam’s topological charge ($\ell$ and/or radial index (*p*), defining the beam’s unique spatial structure.

**Fig. 1:**
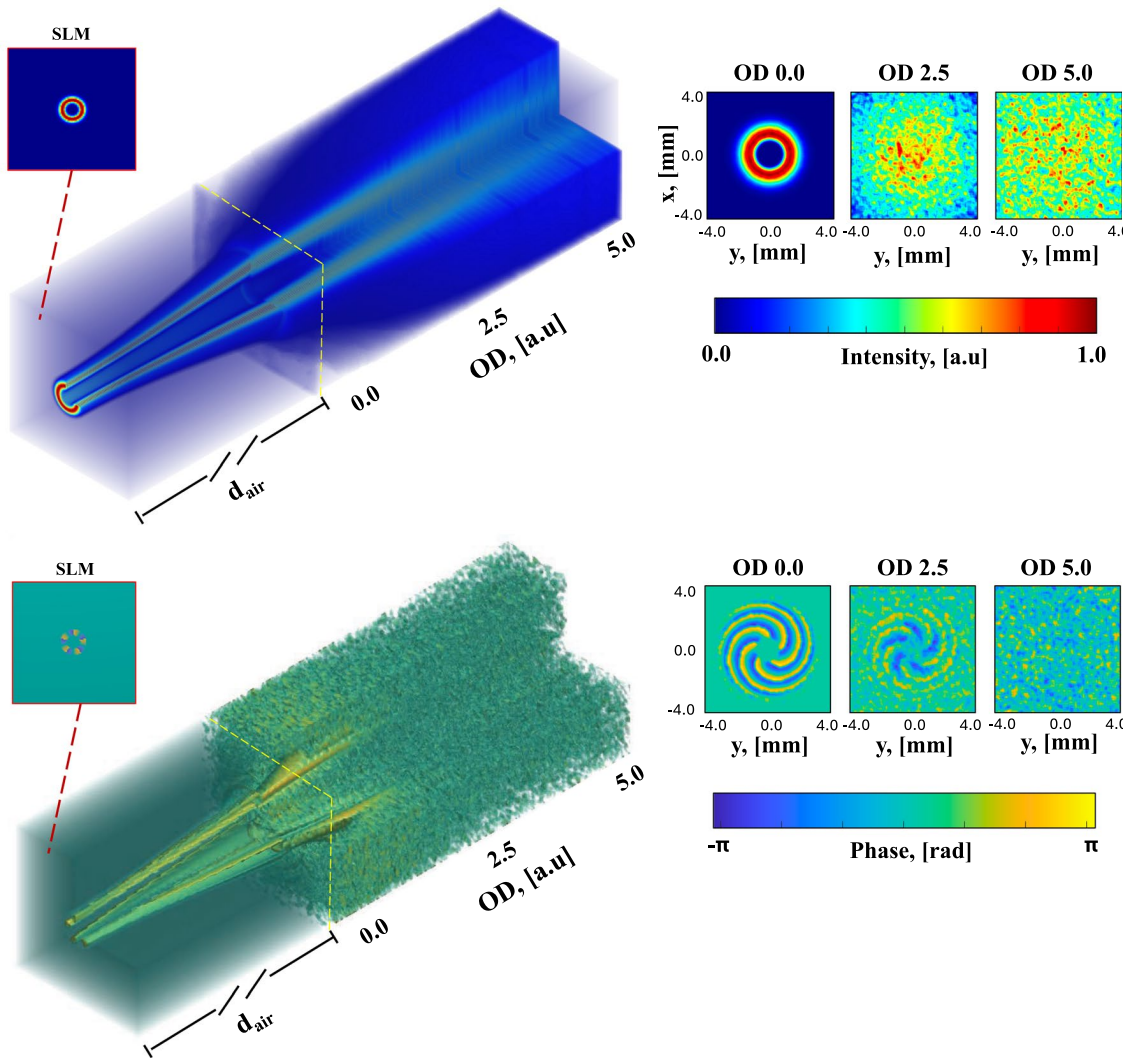
Intensity (top) and phase (bottom) distributions of the $LG_0^5$ beam along propagation from Spatial Light Modulator (SLM) into the complex scattering medium. Insets show, respectively, 2D spatial distributions of intensity (normalized) and phase at the surface of the medium ($d/l^* = 0$) and within the medium at the depth $d/l^* = 2.5$ and $d/l^* = 5.0$.

The results of computational MC modeling shows that during transmission, the beam not only loses intensity due to phenomena like scattering, refraction, and absorption but also experiences beam spreading (Fig. 1). As one can see the speckle interference pattern, resulting from the superposition of helical wavefront components, exhibits spatial variations both in intensity and phase distributions. Such patterns lead to the distortion of the LG beam’s doughnut structure even in low-scattering ($d/l^* = 2.5$) environments (see Fig. 1-(top)) and its complete breakdown in environments characterized by higher scattering ($d/l^* = 5$).

Here, the complex turbid media, distinguished by either low or multiple light scattering, are defined by their optical depth ($d/l^*$), which quantitatively represents the degree of light attenuation and scattering intensity as it travels through the medium; where, *d* denotes the medium’s thickness or the depth of the beam penetration, and $l^*$ is the transport mean free path ($l^* = 1/\mu _s$, where $\mu _s$ is the scattering coefficient of the medium). It is generally accepted that $d/l^*$ of 5–6 or greater indicates a medium undergoing multiple or diffuse scattering, while values between approximately 2 to 5 suggest single or intermediate scattering, characterized as ‘snake-like photons’^26^.

**Fig. 2:**
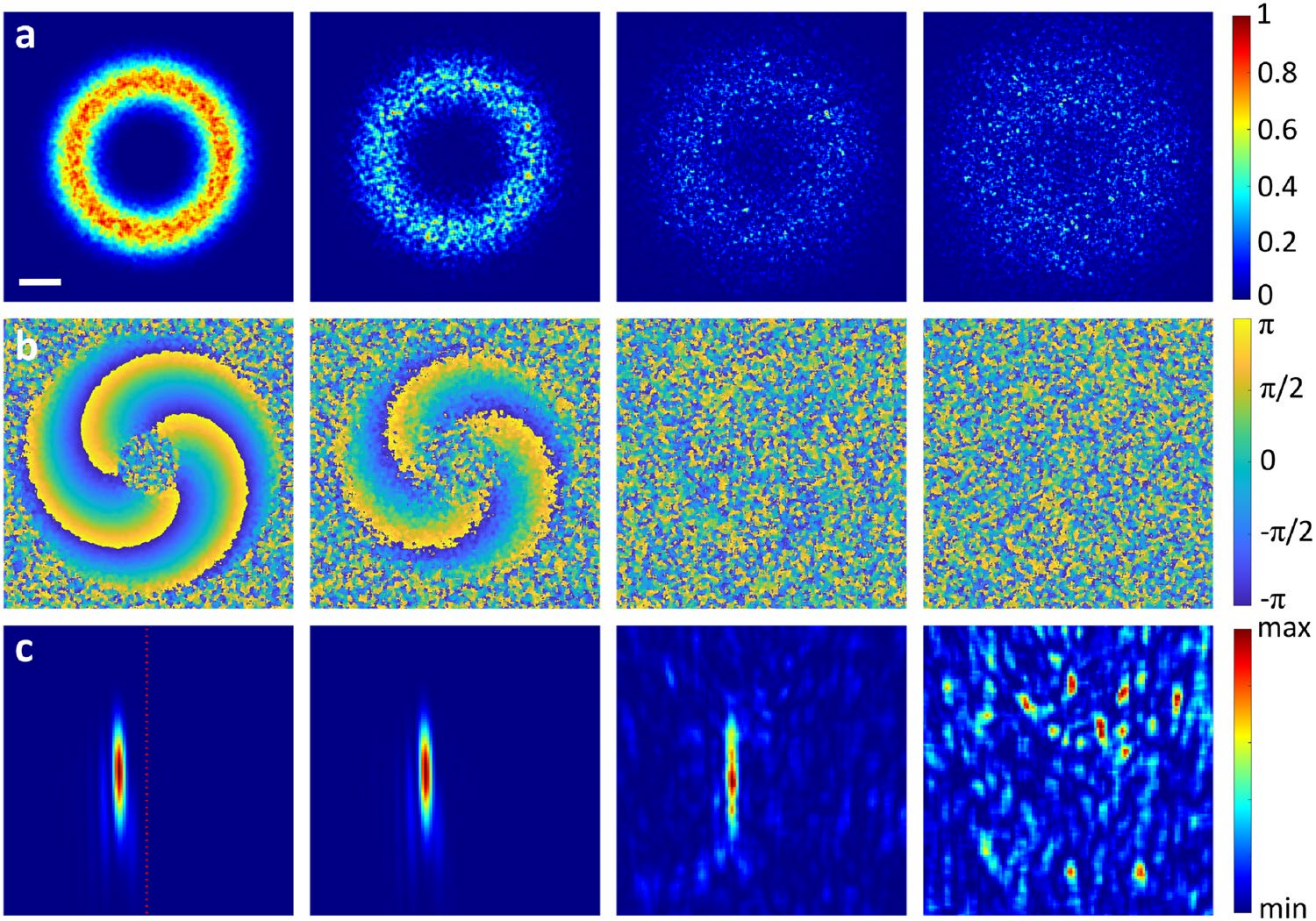
Intensity (**a**) and phase (**b**) distributions of the $LG_0^3$ beam after propagation through a complex scattering medium with 1 mm thickness and specified scattering coefficients $\mu _s=2,4,6,10~\text {mm}^{-1}$; absorption coefficient $\mu _a=0.01~\text {mm}^{-1}$ and anisotropy factor $g=0.8$. Row (**c**) depicts intensity output of the OAM sorter algorithm adopted from^27^. Dotted red line on left figure indicates position of the intensity peak which corresponds to the Gaussian beam without OAM^27^. The scale bar in (**a**) represents 0.5 mm, which applies to all images.

In contrast, in medium with low scattering ($d/l^* \sim 2$), the phase of LG beam progresses with minimal distortion, preserving its original OAM state, petal-like helical phase structure (see Fig. 1-(bottom). Conversely, in higher scattering ($d/l^* = 5$), the LG beam’s phase structure diffusively spreads, and its helical phase front is disrupted, leading to the formation of a complex phase speckle pattern (refer to Fig. 2).

The numerical OAM sorter employed to confirm presence of the topological charge in the scattered signal is adopted from^27^. It simulates optical elements that, firstly, transform the attenuated helical light into beam with a transverse phase gradient and, secondly, focus the OAM state present in the resulting beam to a specific lateral position defined by the value of topological charge $\ell$. This method, originally introduced and experimentally confirmed to efficiently distinguish between OAM states with different charges, exhibits certain potential to detect OAM presence in biomedical diagnostic applications.

Figure 2 demonstrates that the numerical OAM sorter^27^ can detect the OAM memory—the retention of the helical phase structure by the LG beam as it propagates through the scattering medium. More specifically we apply a method for efficiently OAM states of light by employing two static optical elements that transform the helical phase structure of OAM beams into a linear phase gradient^27^. This transformation is achieved through a Cartesian to log-polar coordinate mapping, followed by a phase correction to eliminate distortions. The transformed LG beams are then focused to distinct lateral positions corresponding to their OAM states. The horizontal position of the intensity blob(s) on the detector plane (see Fig. 2) is a direct indicator of the OAM state of the detected light. Each unique OAM state results in a distinct and predictable lateral position, allowing for efficient sorting and detection of multiple OAM states simultaneously.

The preservation of the OAM phase, known as OAM phase memory, is ascribed to the congruence in the alterations of the spiral trajectories encircling the beam axis, arising from rotational symmetry. The spiral trajectories undergo substantial modifications from their initial LG beam configurations due to the interaction with the scattering medium. This leads to disparate partial components of the LG beam’s helical wavefront undergoing varying extents of phase distortions. Owing to the inherent rotational symmetry, these alterations in the spiral trajectories exhibit uniformity in both magnitude and direction around the beam’s axis. Consequently, despite the significant phase distortions resulting from multiple scattering events ($d/l^* \sim 10$), the overall retention of the LG beam’s OAM is distinctly evident in the phase behavior of the speckle pattern. This pattern mirrors the modulation of the LG beam’s initial phase as configured by the SLM (see the Supplementary Video).

**Fig. 3:**
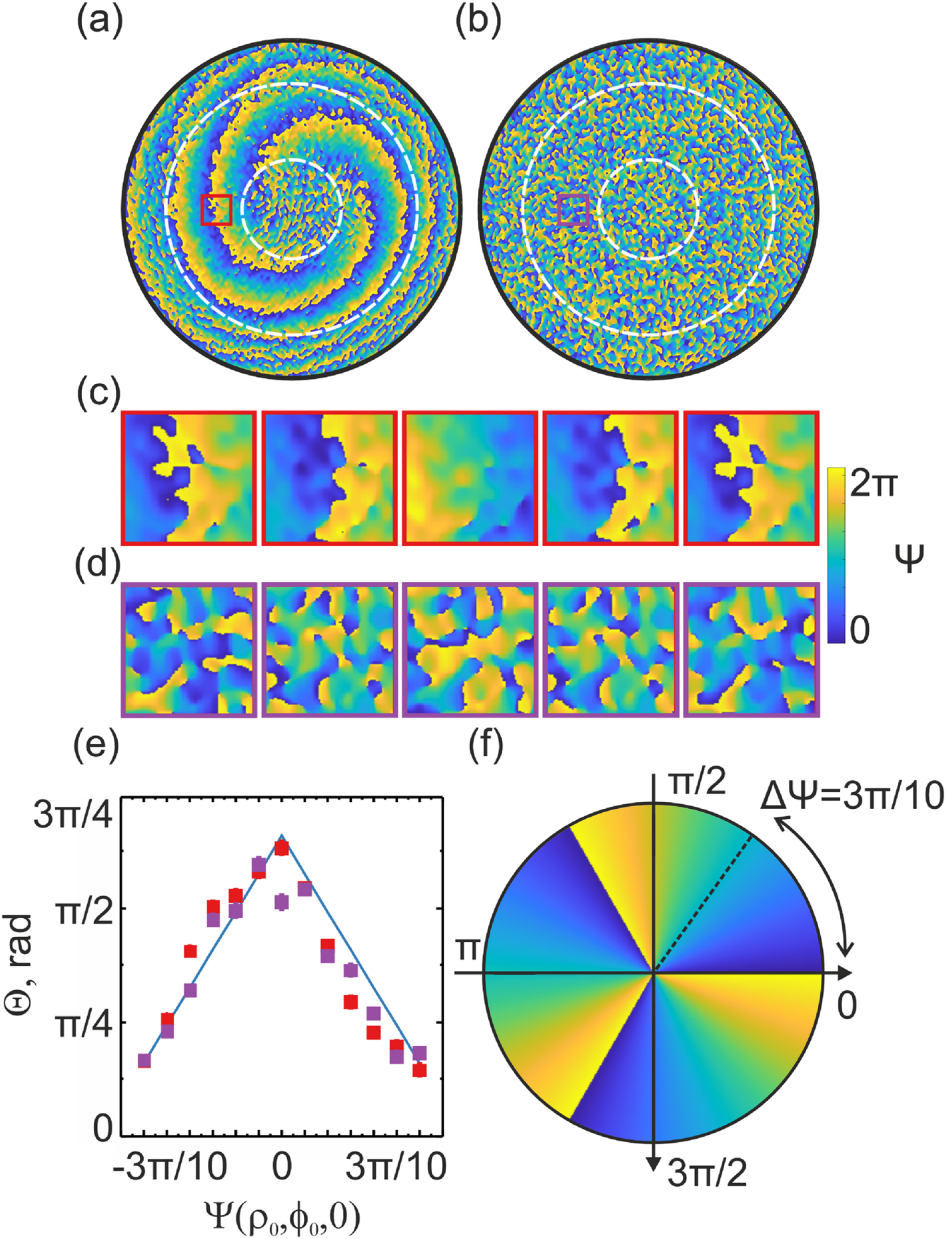
Experimentally observed phase distributions for the LG beam propagating through (**a**) low-scattering ($d/l^* = 2$) and (**b**) multiple-scattering ($d/l^* = 9.6$) media. The central annular zone (highlighted by contours) corresponds to the $LG_0^3$ beam as if it were passing through a non-scattering medium. Phase change at the selected area of the $LG_0^3$ axial annular area for low-scattering (**c**), multiple-scattering (**d**) media and a comparison of relative phase change (**e**) according to the initial phase configuration ($-3\pi /10 \leqslant \Psi \leqslant 3\pi /10$) at the SLM (**f**).

The results of this phenomenological model are well agreed with the experimental results^28^, presented in Fig. 3. The LG beam preserves its initial phase structure and spatial intensity profile, demonstrating its ability to maintain phase coherence and OAM characteristics in a medium with moderate ($d/l^* = 2$) scattering properties (see Fig. 3a). Propagation through the multiple-scattering medium ($d/l^* \sim 10$) caused a diffusive spread and disruption of the LG beam’s helical phase front, resulting in a complex speckle pattern (see Fig. 3b). Despite significant phase distortions, the phase speckle pattern retained initial modulation, indicating partial OAM memory, especially within the axial annular region (see Fig. 3c,d, respectively).

Thus, despite the prevalence of strong diffuse scattering, the phase within the speckle pattern retains modulation reflective of the LG beam’s initial phase, illustrating the persistence of OAM’s memory effect amidst multiple scattering.

## Discussion

This study embarked on an exploration of the propagation dynamics of LG vortex beams carrying OAM through turbid, complex scattering media. The results presented above illustrate the impact of scattering on the phase preservation of an LG beam as it propagates through different scattering media. In the low-scattering medium ($d/l^* = 2$), the LG beam maintains its helical phase structure, with minimal disruption to its initial phase and intensity profile (see Fig. 3a). This demonstrates that the LG beam can retain its OAM characteristics and phase coherence in environments with moderate scattering properties. Conversely, in the multiple-scattering medium ($d/l^* = 9.6$), the LG beam undergoes significant phase distortion, resulting in a complex speckle pattern (see Fig. 3b). Despite these distortions, the speckle pattern retains some modulation of the initial phase, indicating a degree of OAM memory. This partial preservation of the OAM phase is particularly noticeable within the axial annular region, while it diminishes in the central and outer regions beyond this annular zone (see Fig. 3).

Utilizing both experimental methods and in-house developed MC simulations, we investigated the conservation of OAM and the accompanying phase shifts, indicative of the unique ‘twist of light’ phenomena through these challenging environments. Our findings demonstrate a robust preservation of OAM despite the multiple scattering processes, underpinning the potential of OAM for enhanced sensing capabilities in optical communications and remote sensing. The employment of a numerical OAM sorter further validated the persistence of OAM in scattered signals, affirming the foundational concept of OAM phase memory within the scattering media.

**Fig. 4:**
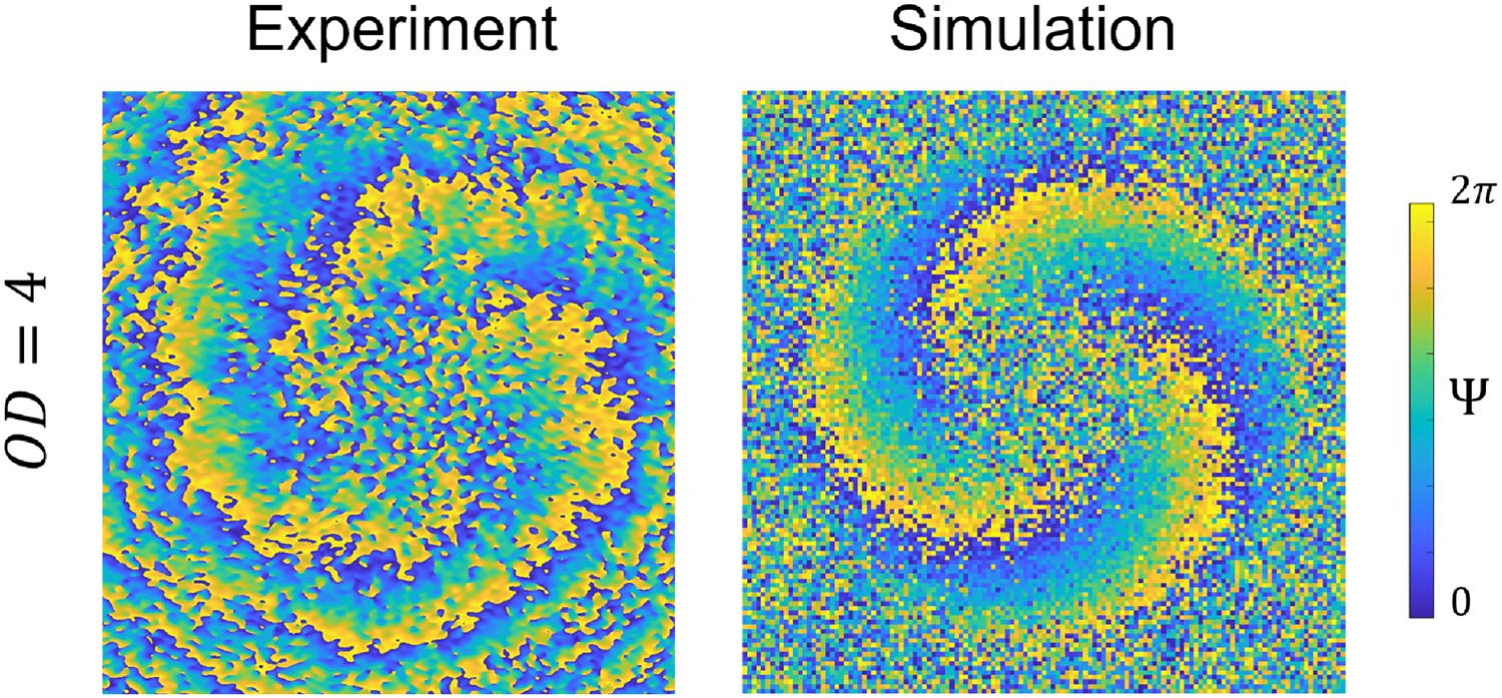
Comparison between simulated and experimentally measured phase patterns for the $LG_0^3$ beam propagated through a complex scattering medium $\mu _s=4~\text {mm}^{-1}$, $\mu _a=0.05~\text {mm}^{-1}$, $g=0.8$.

The investigation reveals that despite the inherent scattering challenges presented by complex media, OAM’s unique properties remain largely intact, exhibiting only minor distortions even in high-scattering environments. This resilience of OAM through turbid media opens new avenues for advanced sensing applications, offering a novel paradigm for optical communication systems that require high penetration depths and robust signal integrity. The successful implementation of MC simulations aligns closely with experimental observations (Fig. 4), providing a powerful tool for predicting light propagation behaviors in similarly complex environments. This study contributes to the fundamental understanding of light-matter interactions within turbid medium, as well as paves the way for the practical application of OAM in diverse fields such as biomedical imaging, atmospheric sensing, and secure communications. Future work will aim to refine the predictive capabilities of our models and explore the potential of multiplexed OAM states for even more complex communication and sensing tasks, thereby leveraging the full spectrum of opportunities afforded by the unique properties of structured light beams.

## Methodology

### Experimental setup

**Fig. 5:**
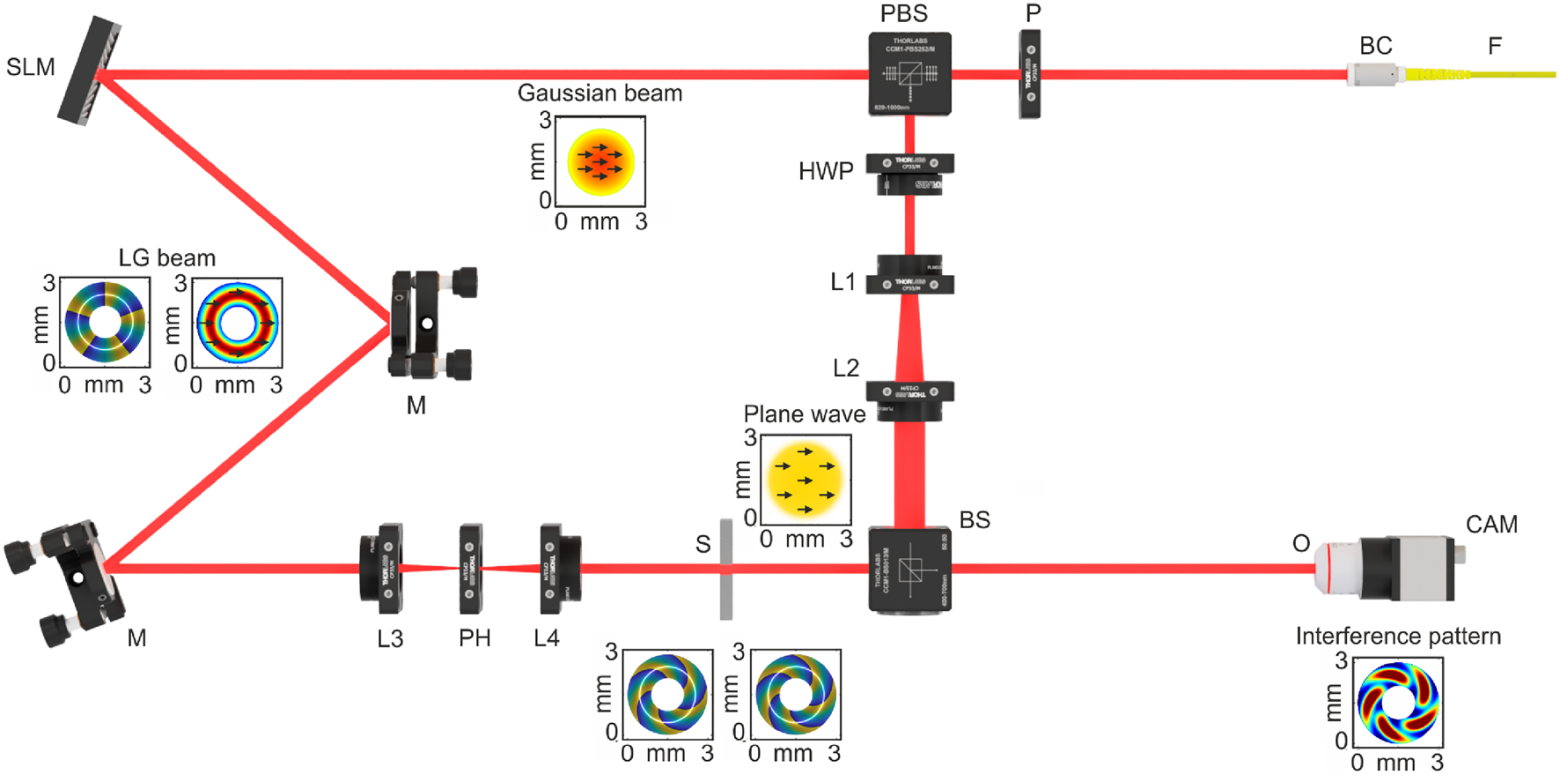
LD—laser diode. P—polarizer. FM—fiber mount. BC—beam colimator. SLM—spatial light modulator. M1, M2—mirrors. L1, L2, L3, L4—lenses. PH—pinhole. PBS—polarizing beam splitter. HWP—half wave plate. S—cuvette filled with the sample liquid. BS—beam splitter. NF—neutral filter. O—objective. CCD—camera. The detailed description of the optical setup is presented in the main article text.

A Mach–Zehnderinterferometer^29,30^, typically used to characterize the relative phase shift between two laser beams, allows to observe the phase structure of the LG beam interfered with the reference plane wave of the same frequency^31^. In this study, the modified Mach–Zehnder-based interferometer^32^ is used to examine an evolution of OAM of the LG beams propagated through the medium (see Fig.5).

In the experiment the coherent laser source (60 mW, OBIS, Coherent, USA) emitting at 633 nm is used as a light source. To clear the optical mode, laser beam was focused into a single-mode optical fiber (Thorlabs, USA) F. The output beam was collimated using beam collimator (Thorlabs, USA) BC to obtain Gaussian beam with 1.6 mm waist diameter. Also, to obtain the horizontal linear polarization the polarizer (Thorlabs, USA) P was been used after collimator. Further, the Gaussian beam has been split in sample and reference beams using polarizing beam splitter (Thorlabs, USA) PBS. The sample beam illuminated the phase only spatial light modulator (PLUTO-2-NIR-011, Holoeye, Germany) SLM operating in reflective regime. To produce LG beam with different moments the corresponding forked diffraction patterns were generated at SLM. The light diffracted from SLM has been directed using set of mirrors M to the lens L3 ($f=45\,\,\text {mm}$, Thorlabs, USA). This lens was used to focus the first order diffraction through pinhole (Thorlabs, USA). Further the LG beam was re-collimated using lens L4 ($f=45\,\,\text {mm}$, Thorlabs, USA).

Finally, sample beam was passed through the custom made turbid tissue-like media of low scattering: $d/l^* = 2$ (thickness of $d = 1~\text {mm}$ and $\mu _s = 10~\text {mm}^{-1}$) and multiple-scattering: $d/l^* \sim 10$ (thickness of $d = 8~\text {mm}$ and $\mu _s = 6~\text {mm}^{-1}$). The anisotropy of scattering in both cases was $g = 0.8$. The detailed methodology for the preparation of such phantoms is thoroughly described in the study by Sieryi et al., as referenced in^33^. The reference beam was passed through the half-wave plate (Thorlabs, USA) to control the polarization orientation of Gaussian beam. Further, reference beam was expanded by set of lenses L1 ($f=30\,\,\text {mm}$ Thorlabs, USA) and L2 ($f=70\,\,\text {mm}$, Thorlabs, USA) and directed to the beam splitter (Thorlabs, USA) BS where expanded Gaussian beam interfered with LG beam. Finally, the interference pattern was registered using CMOS camera (DCC3240M, $1280 \times 1024$, Thorlabs, USA) CAM in combination with objective (10$\times$, Nikon, Japan) O. The proposed setup allowed to obtain interference patterns in both on-axis^34^ and off-axis^35^ regimes. The corresponding simulated intensity profiles for Gaussian beam, expanded Gaussian beam, $LG^5_0$ beam and corresponding interference pattern for on-axis regime are shown in “jet” colour palette at Fig.5. Black arrows shows the polarization direction. The corresponding simulated phases for $LG^5_0$ beam at the SLM, before the cuvette with sample liquid and after the cuvette with sample liquid are show in “parula” colour palette at Fig. 5.

### Simulation

MC method is vastly used as an effective tool for imitation of image transfer through turbid scattering media^36^, including complex structured ones like biological tissues^37^ where the technique is considered as ‘gold standard’^38,39^. Recently, a number of various MC methods have been developed to simulate propagation of polarized light within turbid tissue-like scattering media^40–43^. The MC approach, informed by the exact analytic Milne solution and utilizing the iterative solution to the Bethe–Salpeter equation^44–46^ with Jones vector formalism, effectively tracks the polarization of MC-photons within turbid tissue-like media and simulates coherent backscattering^47,48^. Advantages of the Bethe–Salpeter-based approach involve a direct relation to the analytic Milne solution^49^ and intuitive physical interpretation of multiple scattering process via ladder diagrams. The approach has subsequently progressed to include polarized light, extending the computational capabilities of biomedical optics diagnostic toolbox^50^. Fundamental ground for the polarized MC approaches was established by the Vector Radiative Transfer Equation (VRTE) which represents a system of equations for each Stokes parameter and can be rigorously derived from the Maxwell electromagnetic theory^51–53^. Recently, it has been shown on the fundamental level that VRTE and Bethe–Salpeter-based approaches are equivalent under certain conditions^54^. Modern implementations of the polarization-resolved MC^50^ aim to provide a comprehensive description of polarized light scattering with either Jones or Mueller formalism, depending on the representation of the polarization state^55^. Recent biomedical polarimetry advancements highlight the efficacy of circularly polarized light (CPL) in characterizing tissues with optical anisotropy^56–58^. Accurate simulation tools are essential for studying CPL-tissue interactions^59,60^.

The inclusion of shaped light carrying OAM in MC modeling has garnered attention for its potential to enhance the biomedical diagnosis toolkit by offering deeper insights into the structural complexities of turbid tissue-like media and overcoming existing limitations^61–63^. In MC modeling of OAM light, initial phase $\psi _{0_j}$ is determined according to (2). Correspondingly, initial direction is determined utilizing Poynting vector direction ($\vec{p} = \{ p_{\rho } ,p_{\phi } ,p_{z} \}$)^23^:

3 $$\begin{aligned} \begin{aligned}{}&p_{r}= \frac{\omega k r z}{\left( z_{R}^{2}+z^{2}\right) } |LG^\ell _p(r,\phi ,z) |^{2}, \\&p_{\phi }=\frac{\omega l}{r} |LG^\ell _p(r,\phi ,z)|^{2}, \\&p_{z}= \omega k |LG^\ell _p(r,\phi ,z)|^{2} \text{, } \end{aligned} \end{aligned}$$

where $\omega$ is the angular frequency of the light. Regarding the MC method, within the context of corresponding Cartesian coordinates, this is represented by a direction vector ($\vec{s} = \{ s_{x} ,s_{y} ,s_{z} \}$):

$$\begin{aligned} {{s}}_{x}=\frac{k z x}{\left( z^2+z_R^2\right) }-\frac{\ell y}{\left( x^2+y^2\right) }, \quad {{s}}_y=\frac{k z y}{\left( z^2+z_R^2\right) }+\frac{\ell x}{\left( x^2+y^2\right) }, \quad {{s}}_z=k, \end{aligned}$$

As previously above, the polarization tracing framework, rooted in the iterative solution to the Bethe–Salpeter equation and incorporating both Jones and Stokes–Mueller formalisms^50^, effectively models optical phenomena including reflection and refraction for linear, elliptical, and circular polarizations at the medium’s surface. Within the Bethe–Salpeter-based MC model^44,45^, a large amount ($N_{inc}>10^{9}$) of MC-photons with pre-defined statistical weight $W_j, j=[1\ldots N_{inc}]$ is launched from the source oriented under $\theta _i$ angle to the medium surface, propagates through turbid medium and statistics is collected from those $N_{ph} \le N_{inc}$ arrived on the detector. Turbid medium is defined by scattering coefficient $\mu _s$, absorption coefficient $\mu _a$, anisotropy of scattering *g* and refractive index *n*^64^. Each MC-photon defined at the OAM light source is characterized by the initial statistical weight $W_{0_j}$, Cartesian coordinates $(x_{0_j}, y_{0_j}, 0)$, propagation direction $\vec{s}_{0_j}$, initial polarization state and, most importantly, by the initial phase $\psi _{0_j}$. The topological charge of the LG beam determines both $\vec{s}_{0_j}$ and $\psi _{0_j}$. To track the polarization state along the trajectory of the MC-photon, we introduce a real-valued vector $\vec{P}$, representing the direction of the linearly polarized electric field $\vec{E}$^45,46,50^.

After launch, all MC-photons undergo surface ($z=0$) interaction and are transmitted to the turbid medium layer with account for Snell’s law and appropriate Fresnel coefficients influencing MC-photon weights, directions and polarization. In turbid medium ($z>0$) each MC-photon trajectory is modeled as a sequence of the elementary simulations containing limited amount of scattering events $N_{scatt}$. This procedure has been thoroughly covered in previous works^50,64^. At each *i*’th scattering event, $i=[1\ldots N_{scatt}]$, the following computational steps are performed: random path length $l_i=-\textrm{ln}\xi /\mu _s$ is computed (in this paper, we assume that $\mu _a\ll \mu _s$ and $\xi \in (0,1]$ is a uniformly distributed random number), MC-photon is moved to the next position $\vec{r}_{i}  = \vec{r}_{{i - 1}}  + \vec{s}_{i} l_{i}$ with weight attenuated according to the Beer–Lambert law ($W_i=W_{i-1}e^{-\mu _a l_i}$), and next propagation direction $\vec{s}_{i+1}$ is evaluated via inversion of the Henyey–Greenstein (HG) phase function^65^

$$\begin{aligned} p_{HG}(\cos \theta ^{\prime })=\frac{1}{4 \pi } \frac{1-g^2}{\left( 1+g^2-2 g \cos \theta ^{\prime }\right) ^{3 / 2}}, \end{aligned}$$

where $\theta ^{\prime }$ is the polar scattering angle in the MC-photon reference plane. Here, we have used position vector $\vec{r}_{i}  = (x_{i} ,y_{i} ,z_{i} )$ and unit direction for each scattering event $\vec{s}_{i}  = [s_{X} ,s_{Y} ,s_{Z} ]_{i}  = [\sin \theta \;\cos \varphi ,\;\sin \;\theta \;\sin \varphi ,\;\cos \theta ]_{i}$, with $\theta ,\varphi$ as azimuthal and polar angles that correspond to the global Cartesian coordinates. It should be noted that, basically, any phase function *p* can be used^47,66^. If analytical inversion of *p* is not possible, then table lookup method is involved to ensure fast computational speed. At each step we check if MC-photon path crosses the medium boundary and invoke surface refraction-transmission and detection procedures if this is the case. Evolution of each linearly polarized state $\vec{P}_{x}  = \left( {P_{{xx}} ,P_{{xy}} ,P_{{xz}} } \right),$ $\vec{P}_{y}  = \left( {P_{{yx}} ,P_{{yy}} ,P_{{yz}} } \right)$ can be traced along MC-photon trajectory $\vec{r}_{i}$, $i=[1\ldots N_{scatt}]$ via the following procedure which is obtained from the iterative solution to Bethe–Salpeter equation^50,63^:

4 $$ \begin{array}{*{20}l}    {\vec{P}_{i}  =  - \vec{s}_{i}  \times \left[ {\vec{s}_{i}  \times \vec{P}_{{i - 1}} } \right] = \left[ {\hat{I} - \vec{s}_{i}  \otimes \vec{s}_{i} } \right]\vec{P}_{{i - 1}} ,} \hfill  \\   \end{array}  $$

where ${\hat{I}}$ is the third-rank unit tensor and $\otimes$ indicates a direct product.

We repeat outlined computational steps for each scattering event until one of the following conditions is met: either $W_i<10^{-4}$ (statistical weight becomes negligible as follows from Beer–Lambert law) or the amount of scattering events $N_{scatt}$ becomes larger than $10^3$. These limitations ensure proper trajectory tracing cut-off^63^. We continue launching MC-photons until the certain amount (no less than $N_{ph}=10^7$) arrives on the detector. Detection procedure consists of the two checks: MC-photon coordinates should lie within the detector area ($-r_d\le x_N \le r_d, -r_d \le y_N \le r_d, z_N=0$), and refracted direction ${\vec{s}}_N$ should meet the detector numerical aperture (*NA*) requirements. We would limit those directions by using $\textrm{acos}({\textbf{s}}_N \cdot {\textbf{s}}_d)<NA$, where ${\textbf{s}}_d=[\sin (-\theta _d), 0, \cos (-\theta _d)]$ is a unit vector collinear to the detector axis. Both here and in the subsequent sections *N* is considered to be an index of the detection event.

Thus, each detected MC-photon has defined by the following total statistical weight influenced by its path within medium and depolarization:

$$\begin{aligned} W_{N_j} = W_{0_j} \left( P_{xx}^2 + P_{yx}^2 + P_{xy}^2 + P_{yy}^2 \right) _{N_j} \Gamma ^{N_j}_R \exp \left( -\mu _a\sum \limits _{i=1}^{N_j}l_i\right) , \end{aligned}$$

where $0<N_j<N_{scatt}$ is the index of detection event for *j*’th MC-photon, $l_i$ is the path length between two neighbouring scattering events and $\Gamma _R$ is the Rayleigh factor^45,49,63^. Based on our Bathe-Salpeter based polarization tracking approach, we are also able to introduce polarized $W^{\parallel }_{N_j}$ and depolarized $W^{\perp }_{N_j}$ weights for each MC-photon:

5 $$\begin{aligned} W^{\parallel }_{N_j}= &   W_{0_j} \left( P_{xx}^2+P_{yx}^2 \right) _{N_j} \Gamma ^{N_j}_R \exp \left( -\mu _a\sum \limits _{i=1}^{N_j}l_i\right) , \end{aligned}$$

6 $$\begin{aligned} W^{\perp }_{N_j}= &   W_{0_j} \left( P_{xy}^2+P_{yy}^2 \right) _{N_j} \Gamma ^{N_j}_R \exp \left( -\mu _a\sum \limits _{i=1}^{N_j}l_i\right) . \end{aligned}$$

By ensemble averaging within each detector pixel we are able to obtain both intensity and phase values on the detector:

$$\begin{aligned} I_{px}(x,y)= &   \sum _{j=1}^{N_{p x}} W_{N_j}(x,y)+2 \sum _{i=1}^{N_{p x}} \sum _{j>i}^{N_{p x}} \sqrt{W_{N_i}(x,y)} \sqrt{W_{N_j}(x,y)} \cos \big (\Psi _{N_i}(x,y)-\Psi _{N_j}\big (x,y)), \\ \psi (x,y)= &   \arctan \dfrac{\sum \limits _{j=1}^{N_{px}}\sqrt{W_{N_j}(x,y)}\sin {\Psi _{N_j}(x,y)}}{\sum \limits _{j=1}^{N_{px}}\sqrt{W_{N_j}(x,y)}\cos {\Psi _{N_j}(x,y)}}. \end{aligned}$$

Here, we assume that $N_{px}<N_{ph}$ is the amount of photons that arrived at the specific pixel, $\Psi _{N_j}$ is the phase of the *j*-th detected photon, $I_{px}$ is the resulting intensity in this pixel and $\psi (x,y)$ is the resulting phase value in this pixel.

The MC method, a foundational technique in photon transport simulation, has historically encountered the challenge of balancing detailed, accurate modeling with significant computational demands. It has been successfully used for the imitation of image transfer through the complex scattering media, taking into account major experimental parameter, including density, propagation distance, medium optical properties, beam waist, coherence, polarization, etc^36^. The MC is a robust and versatile tool for modeling light propagation through scattering media, valid across various scattering regimes, including Rayleigh ($X/\lambda \ll 1$), Mie ($X/\lambda \approx 1$), and geometrical optics ($X/\lambda \gg 1$) regimes; Here *X* is the smallest dimension of the main scatterer. Renowned for its exceptional precision in representing the intricate interactions between light and biological tissues, the MC method is widely regarded as the ‘gold standard’^39^.

Despite this, the MC method’s reliance on extensive computational resources has been a longstanding issue, with traditional simulations relying heavily on central processing units (CPUs). While the shift from CPU to GPU processing marked a revolutionary decrease in computation time—transforming processes that once spanned hours into mere seconds—this evolution brought with it an increased burden on power consumption and a heightened environmental impact, reflecting a growing concern in our climate-conscious era.

The above mentioned realization of MC implements, for the first time, the energy-effective MC algorithm for complex light propagation utilizing the recently introduced Apple M-family processors^67^. These cutting-edge chips feature a unified memory architecture, which became a revolutionary advancement in our needs of simulating complex light transfer. This development effectively eliminates the previous constraints on the number of photon packets or statistics that need to be stored and processed, dramatically broadening the scope for MC simulations of photon behaviour. Such capability enables the detailed study/statistical analyses of light propagation through turbid media without the computational and environmental costs historically associated with such efforts. In our evaluations, that the efficiency of these processors has been highlighted by their ability to conduct simulations using approximately 100 times less energy than their traditional GPU counterparts and achieve 300 to 10000 times speed increases than those possible with conventional MC simulations.

The M-family chips possess a sophisticated architecture uniquely optimized for parallel processing, a key factor in their superior performance. We utilize the Metal Compute framework developed by Apple, which plays a crucial role in harnessing these capabilities for MC simulations. Metal provides a low-overhead, hardware-accelerated graphics and compute Application Programming Interface (API) finely tuned for parallel data processing, offering a C-style programming language that is both powerful and efficient. Within this framework, compute shaders act as programmable kernel functions, specially designed to execute MC algorithms on the GPU, enabling the detailed and complex simulation of photon trajectories with unparalleled efficiency and speed.

## Supplementary Information

Supplementary Information.

## Data Availability

All data related to the experiments and computational modeling described in this article are archived on a lab computer at Aston University. All data are available from the corresponding author upon reasonable request.

## Code availability

Our models are open-source and also available online as web applications, providing a variety of other MC simulations such as sampling volume, fluence rates, spectra, and colour. The codes used for the modeling of conversion of OAM for different types of LG beams and evaluation the twist of OAM in experimental studies are available from the corresponding author upon reasonable request.

## References

[1] Cao, H., Mosk, A. P. & Rotter, S. Shaping the propagation of light in complex media. *Nat. Phys.* **18**(9), 994–1007 (2022). 10.1038/s41567-022-01677-x

[2] Mishchenko, M. I., Travis, L. D. & Lacis, A. A. *Multiple Scattering of Light by Particles: Radiative Transfer and Coherent Backscattering* (Cambridge University Press, Cambridge, 2006).

[3] Choy, T. C. *Effective Medium Theory* (Oxford University Press, Oxford, 2016).

[4] Wenshan, C. & Shalaev, V. *Optical Metamaterials: Fundamentals and Application* (Springer, New York, 2009).

[5] Chandrasekhar, S. *Radiative Transfer* (Oxford University Press, New York, 1950).

[6] Ishimaru, A. *Wave Propagation and Scattering in Random Media, vol I and II* (Academic, New York, 1978).

[7] Lu, B., Morgan, S. P., Crowe, J. A. & Stockford, I. M. Comparison of methods for reducing the effects of scattering in spectrophotometry. *Appl. Spectrosc.* **60**, 1157–66 (2006). 17059668 10.1366/000370206778664725

[8] Lee, H. *et al.* High-throughput volumetric adaptive optical imaging using compressed time-reversal matrix. *Light Sci. Appl.* **11**, 16 (2022). 35027538 10.1038/s41377-021-00705-4 PMC8758712

[9] Sanjeev, A. *et al.* Non-invasive imaging through scattering medium by using a reverse response wavefront shaping technique. *Sci. Rep.* **10**, 6029 (2020). 32238830 10.1038/s41598-020-62442-9 PMC7113255

[10] Sanjeev, A., Trivedi, V. & Zalevsky, Z. Optical reciprocity induced wavefront shaping for axial and lateral shifting of focus through a scattering medium. *Sci. Rep.* **11**, 6387 (2022). 10.1038/s41598-022-10378-7 PMC9013373 35430597

[11] ...Gigan, S. *et al.* Roadmap on wavefront shaping and deep imaging in complex media. *J. Phys. Photon.* **4**(4), 042501 (2022). 10.1088/2515-7647/ac76f9

[12] Yu, Z. *et al.* Wavefront shaping: A versatile tool to conquer multiple scattering in multidisciplinary fields. *Innovation (Cambridge)* **3**, 100292 (2022). 10.1016/j.xinn.2022.100292 PMC9405113 36032195

[13] Shen, Y. *et al.* Optical vortices 30 years on: OAM manipulation from topological charge to multiple singularities. *Light Sci. Appl.* **8**, 90 (2019). 31645934 10.1038/s41377-019-0194-2 PMC6804826

[14] He, C., Shen, Y. & Forbes, A. Towards higher-dimensional structured light. *Light Sci. Appl.* **11**, 205 (2022). 35790711 10.1038/s41377-022-00897-3 PMC9256673

[15] Cao, H., Čižmár, T., Turtaev, S., Tyc, T. & Rotter, S. Controlling light propagation in multimode fibers for imaging, spectroscopy, and beyond. *Adv. Opt. Photon.* **15**(2), 524–612 (2023). 10.1364/AOP.484298

[16] Wang, W. B., Gozali, R., Shi, L., Lindwasser, L. & Alfano, R. R. Deep transmission of Laguerre–Gaussian vortex beams through turbid scattering media. *Opt. Lett.* **41**(9), 2069–2072 (2016). 27128076 10.1364/OL.41.002069

[17] Angelsky, O. V., Mokhun, I. I., Bekshaev, A. Y., Zenkova, C. Y. & Zheng, J. Polarization singularities: Topological and dynamical aspects. *Front. Phys.* **11**, 1147788 (2023). 10.3389/fphy.2023.1147788

[18] Forbes, A. Advances in orbital angular momentum lasers. *J. Light. Technol.* **41**(7), 2079–2086 (2023). 10.1109/JLT.2022.3220509

[19] Mamani, S. *et al.* OAM transmission of polarized multipole laser beams in rat cerebellum tissue. *Opt. Commun.* **532**, 129241 (2023). 10.1016/j.optcom.2022.129241

[20] Biton, N., Kupferman, J. & Arnon, S. OAM light propagation through tissue. *Sci. Rep.* **11**(1), 2407 (2021). 33510283 10.1038/s41598-021-82033-6 PMC7843596

[21] Wang, W. B. *et al.* Optical vortex beam transmission with different OAM in scattering beads and brain tissue media. In *Complex Light and Optical Forces X*, Vol. 9764 114–119 (2016).

[22] Wang, Y. *et al.* Orbital angular momentum of Laguerre–Gaussian beams with non-zero radial index at limited aperture size. *Results Phys.* **48**, 106436 (2023). 10.1016/j.rinp.2023.106436

[23] Allen, L., Padgett, M. J. & Babiker, M. *The Orbital Angular Momentum of Light* Vol. 39, 291–372 (Elsevier, North-Holland, 1999).

[24] Rosales-Guzmán, C., Ndagano, B. & Forbes, A. A review of complex vector light fields and their applications. *J. Opt.* **20**(12), 123001 (2018). 10.1088/2040-8986/aaeb7d

[25] Berry, M. V. & McDonald, K. T. Exact and geometrical optics energy trajectories in twisted beams. *J. Opt. A Pure Appl. Opt.* **10**(3), 035005 (2008). 10.1088/1464-4258/10/3/035005

[26] Yoo, K. M., Liu, F. & Alfano, R. R. When does the diffusion approximation fail to describe photon transport in random media?. *Phys. Rev. Lett.* **64**, 2647–2650 (1990). 10041774 10.1103/PhysRevLett.64.2647

[27] Berkhout, G. C. G., Lavery, M. P. J., Courtial, J., Beijersbergen, M. W. & Padgett, M. J. Efficient sorting of orbital angular momentum states of light. *Phys. Rev. Lett.* **105**, 153601 (2010). 21230900 10.1103/PhysRevLett.105.153601

[28] Meglinski, I., Sdobnov, A., Lopushenko, I. & Bykov, A. Phase memory of orbital angular momentum in multiple scattering environment. *Laser Sci. Appl.* (arXiv preprint arXiv:2312.08928) (2024–in press) 10.1038/s41377-024-01562-7 PMC11347564 39187516

[29] Mach, L. Ein neuer Interferenzrefraktor. *Z. Instrum.* **12**, 89–93 (1892).

[30] Zehnder, L. Ein neuer Interferenzrefraktor. *Z. Instrum.* **11**, 275–285 (1891).

[31] Padgett, M., Arlt, J., Simpson, N. & Allen, L. An experiment to observe the intensity and phase structure of Laguerre–Gaussian laser modes. *Am. J. Phys.* **64**(1), 77–82 (1996). 10.1119/1.18283

[32] Kumar, P. & Nishchal, N. K. Modified Mach–Zehnder interferometer for determining the high-order topological charge of Laguerre-Gaussian vortex beams. *J. Opt. Soc. Am. A* **36**(8), 1447–1455 (2019). 10.1364/JOSAA.36.001447 31503573

[33] Sieryi, O., Popov, A., Kalchenko, V., Bykov, A. & Meglinski, I. Tissue-mimicking phantoms for biomedical applications. *Proc. SPIE* **11363**, 1136312 (2020).

[34] Cui, S. *et al.* Determining topological charge based on an improved fizeau interferometer. *Opt. Expr.* **27**(9), 12774–12779 (2019). 10.1364/OE.27.012774 31052813

[35] Vayalamkuzhi, P. *et al.* Transform-based phase retrieval techniques from a single off-axis interferogram. *Appl. Opt.* **60**(19), 5523–5533 (2021). 34263840 10.1364/AO.422900

[36] Berrocal, E., Meglinski, I., Greenhalgh, D. A. & Linne, M. A. Image transfer through the complex scattering turbid media. *Laser Phys. Lett.* **3**(9), 464–468 (2006). 10.1002/lapl.200610035

[37] Carles, G., Zammit, P. & Harvey, A. R. Holistic Monte–Carlo optical modelling of biological imaging. *Sci. Rep.* **9**, 15832 (2019). 31676825 10.1038/s41598-019-51850-1 PMC6825179

[38] Zhu, R., Avsievich, T., Popov, A. & Meglinski, I. Optical tweezers in the studies of red blood cells. *Cells* **9**(3), 545 (2020). 32111018 10.3390/cells9030545 PMC7140472

[39] Periyasamy, V. & Pramanik, M. Advances in Monte Carlo simulation for light propagation in tissue. *IEEE Rev. Biomed. Eng.* **10**, 122–135 (2017). 28816674 10.1109/RBME.2017.2739801

[40] Xu, M. Electric field Monte Carlo simulation of polarized light propagation in turbid media. *Opt. Express* **12**, 6530–6539 (2004). 19488304 10.1364/OPEX.12.006530

[41] Gangnus, S. V., Matcher, S. J. & Meglinski, I. Monte Carlo modeling of polarized light propagation in biological tissues. *Laser Phys.* **14**(6), 886–891 (2004).

[42] Ramella-Roman, J. C., Prahl, S. A. & Jacques, S. L. Three Monte Carlo programs of polarized light transport into scattering media: Part I. *Opt. Express* **13**(12), 4420–4438 (2005). 19495358 10.1364/OPEX.13.004420

[43] Ramella-Roman, J. C., Prahl, S. A. & Jacques, S. L. Three Monte Carlo programs of polarized light transport into scattering media: part II. *Opt. Express* **13**(25), 10392–10405 (2005). 19503254 10.1364/OPEX.13.010392

[44] Kuzmin, V. L. & Meglinski, I. V. Coherent multiple scattering effects and Monte Carlo method. *JETP Lett.* **79**, 109–112 (2004). 10.1134/1.1719124

[45] Meglinski, I., Kuzmin, V. L., Churmakov, D. Y. & Greenhalgh, D. A. Monte Carlo simulation of coherent effects in multiple scattering. *Proc. R. Soc. A* **463**, 43–53 (2005). 10.1098/rspa.2004.1369

[46] Kuzmin, V. L. & Meglinski, I. V. Coherent effects of multiple scattering for scalar and electromagnetic fields: Monte–Carlo simulation and Milne-like solutions. *Opt. Commun.* **273**(2), 307–310 (2007). 10.1016/j.optcom.2007.01.025

[47] Doronin, A., Radosevich, A. J., Backman, V. & Meglinski, I. Two electric field Monte Carlo models of coherent backscattering of polarized light. *J. Opt. Soc. Am. A* **31**(11), 2394–2400 (2014). 10.1364/JOSAA.31.002394 25401350

[48] Meglinski, I. & Kuz’min, V. L. Coherent backscattering of circularly polarized optical radiation from a disperse random medium. *Prog. Electromagn. Res. M* **21**, 1972–1977 (2011).

[49] Akkermans, E., Wolf, P. E., Maynard, R. & Maret, G. Theoretical study of the coherent backscattering of light by disordered media. *J. Phys. France* **49**, 77–98 (1988). 10.1051/jphys:0198800490107700

[50] Lopushenko, I., Sieryi, O., Bykov, A. & Meglinski, I. Exploring the evolution of circular polarized light backscattered from turbid tissue-like disperse medium utilizing generalized Monte Carlo modeling approach with a combined use of Jones and Stokes–Mueller formalisms. *J. Biomed. Opt.* **29**, 052913 (2024). 38089555 10.1117/1.JBO.29.5.052913 PMC10715447

[51] Mishchenko, M. I. Vector radiative transfer equation for arbitrarily shaped and arbitrarily oriented particles: A microphysical derivation from statistical electromagnetics. *Appl. Opt.* **41**, 7114–7134 (2002). 12463259 10.1364/AO.41.007114

[52] Raković, M. J. *et al.* Light backscattering polarization patterns from turbid media: Theory and experiment. *Appl. Opt.* **38**, 3399–3408 (1999). 18319938 10.1364/AO.38.003399

[53] Tynes, H. H. *et al.* Monte Carlo and multicomponent approximation methods for vector radiative transfer by use of effective Mueller matrix calculations. *Appl. Opt.* **40**, 400–412 (2001). 18357013 10.1364/AO.40.000400

[54] Doicu, A. & Mishchenko, M. I. An overview of methods for deriving the radiative transfer theory from the Maxwell equations. II: Approach based on the Dyson and Bethe–Salpeter equations. *J. Quant. Spectrosc. Radiat. Transf.* **224**, 25–36 (2019). 30713354 10.1016/j.jqsrt.2018.10.032 PMC6350797

[55] Günhan Akarçay, H., Hohmann, A., Kienle, A., Frenz, M., & Rička, J.: Monte Carlo modeling of polarized light propagation: Stokes vs. Jones. Part I. *Appl. Opt.* **53**(31), 7576–7585 (2014). 10.1364/AO.53.007576 25402926

[56] Ivanov, D. *et al.* Colon cancer detection via Poincaré sphere representation and 2D polarimetric mapping of ex vivo tissue samples. *J. Biophoton.* **13**, 202000082 (2020). 10.1002/jbio.202000082 32390327

[57] Borovkova, M. A., Bykov, A. V., Popov, A. & Meglinski, I. V. Role of scattering and birefringence in phase retardation revealed by locus of Stokes vector on Poincaré sphere. *J. Biomed. Opt.* **25**(5), 057001 (2020). 32436372 10.1117/1.JBO.25.5.057001 PMC7238295

[58] Singh, M. D. & Vitkin, I. A. Spatial helicity response metric to quantify particle size and turbidity of heterogeneous media through circular polarization imaging. *Sci. Rep.* **13**(1), 2231 (2023). 36755076 10.1038/s41598-023-29444-9 PMC9908950

[59] Nishizawa, N. & Kuchimaru, T. Depth estimation of tumor invasion in early gastric cancer using scattering of circularly polarized light: Monte carlo simulation study. *J. Biophoton.* **15**(10), 202200062 (2022). 10.1002/jbio.202200062 35666013

[60] Lopushenko, I., Bykov, A. & Meglinski, I. Depolarization composition of backscattered circularly polarized light. *Phys. Rev. A* **108**, 041502 (2023). 10.1103/PhysRevA.108.L041502

[61] Doronin, A., Milione, G., Meglinski, I. & Alfano, R. R. Propagation and scattering of vector light beam in turbid scattering medium. *Proc. SPIE* **8940**, 894006 (2014). 10.1117/12.2038818

[62] Doronin, A., Vera, N., Staforelli, J.P., Coelho, P. & Meglinski, I. Propagation of cylindrical vector laser beams in turbid tissue-like scattering media. Photonics **6**(2), 56–67 (2019)

[63] Doronin, A., Novikova, V. N. Tatiana, Staforelli, J. P. & Meglinski, I. Assessment of twisted light localization in turbid tissue-like scattering media using 3D geometrical exploration. In *Proceedings of SPIE PC12373* 1237308 (2023).

[64] Meglinski, I. & Doronin, A. Chapter 1. In *Advanced Biophotonics: Tissue Optical Sectioning* (eds Wang, R. K. & Tuchin, V. V.) 1–72 (CRC Press, Boca Raton, 2013).

[65] Henyey, L. G. & Greenstein, J. L. Diffuse radiation in the galaxy. *Astrophys. J.* **93**, 70–83 (1941). 10.1086/144246

[66] Kuz’min, V. L., Val’kov, A. Y. & Zubkov, L. A. Photon diffusion in random media and anisotropy of scattering in the Henyey–Greenstein and Rayleigh–Gans models. *J. Exp. Theor. Phys.* **128**, 396–406 (2019). 10.1134/S1063776119020109

[67] Clennell, A., Nguyen, V., Yakovlev, V. S. & Doronin, A. Neu(t)ralMC: Energy-efficient open source Monte Carlo algorithm for assessing photon transport in turbid media. *Opt. Express* **31**(19), 30921–30931 (2023). 37710624 10.1364/OE.496516 PMC10544956

